# Battling illness with wellness: a qualitative case study of a young rapper’s experiences with music therapy

**DOI:** 10.1080/08098131.2014.907334

**Published:** 2014-05-01

**Authors:** Hans Petter Solli

**Affiliations:** ^a^ Lovisenberg Diakonale Hospital, Oslo, Norway; ^b^ GAMUT, The Grieg Academy, University of Bergen, Bergen, Norway

**Keywords:** music therapy, mental health, psychosis, user perspective, social recovery

## Abstract

Mental health difficulties are connected with major interpersonal and social challenges. Recent qualitative research indicates that music therapy can facilitate many of the core elements found to promote social recovery and social inclusion, findings also reflected in results from a growing body of effect studies. The objective of this study was to explore how music therapy might afford possibilities for social recovery to one man with psychosis admitted to a psychiatric intensive care unit. This was achieved by means of a qualitative case study featuring a description of the music therapeutic process alongside first-hand accounts of the participant’s subjective experiences. The data were analysed using interpretative phenomenological analysis (IPA). The findings are presented in a narrative form reflecting processes and activities considered particularly important for the process of social recovery. Theoretical perspectives from the recovery literature and current perspectives in music therapy are discussed with a view to the possible use of music therapy for strengthening agency, (re)building identity, developing positive relationships, and expanding social networks.

## Introduction

*If I got no music in my head, I’ll be lying like dead in the bed*. This line came from a man who will here be referred to as Marco as he was freestyling^1^ ^1^A style of rap in which lyrics are improvised. to the beats of the drummer in the basement of a psychiatric intensive care unit in Norway. They had a weekly appointment to meet in the music room to play, sing and rap together, and Marco showed up most of the time, except on some days when he could not get out of bed. The drummer was Marco’s music therapist, who is also the author of the present text. This single case study sets out to explore how music therapy might promote recovery and social inclusion for a man admitted to a psychiatric intensive care unit with psychotic experiences. That is, how Marco used music to get out of that bed and into hope-filled social relationships and contexts.

Within the medical setting of the intensive care unit, Marco’s withdrawal to his room and bed was referred to as negative symptoms, a cluster of symptoms including lack of motivation, impaired emotional responsiveness, and asociality. From a biomedical perspective, these conditions are related to neurobiological deficits and cognitive impairment, and hence are treated pharmacologically (Kirkpatrick, Fenton, Carpenter, & Marder, 2006). The medical rationale of negative symptomology has been criticised for ignoring the role of subjective experiences and contextual factors (Slade, 2009). When people with first-hand experiences of mental health problems are asked, they offer alternative ways of understanding their lethargic condition and the isolation. Deegan (1996a) regards “negative symptoms” as necessary strategies on the part of users to survive from experiences of hopelessness. Boevink (2012) explains how she *uses* rigid routines and limited social contact as strategies to avoid overwhelming experiences that can trigger her psychosis. Furthermore, “negative symptoms” are understood as a self-protective response to processes of stigma and social exclusion (Markowitz, 2005; Repper & Perkins, 2003; Strauss, 1989), and to demoralisation caused by paternalistic and pathological treatment systems (Topor, Borg, Di Girolamo, & Davidson, 2011).

Negative symptoms are found to be a predictor of poor functioning and reduced quality of life (Katschnig, 2000). Mental health difficulties in general are associated with reduced social networks (Repper & Perkins, 2003) which also increase mortality risk (Holt-Lunstad, Smith, Layton, & Brayne, 2010). Longstanding efforts to develop effective medical treatment to reduce negative symptoms have produced limited results (Kirkpatrick et al., 2006). However, music therapy studies have recently demonstrated positive effects on negative symptoms and social functioning for patients with severe mental illness (Gold et al., 2013; Gold, Solli, Krüger, & Lie, 2009; Mössler, Chen, Heldal, & Gold, 2011). Other research indicates that sociability and satisfying social networks are important factors as they are associated with enhanced hope (Adams & Partee, 1998; Byrne et al., 1994), well-being (Jetten, Haslam, Haslam, Dingle, & Jones, 2014; Kogstad, Mönness, & Sörensen, 2013; Ponde, Peireira, Leal, & Oliveira, 2009), and quality of life (Hansson, 2006). The World Health Organization’s (WHO) review of the evidence for social determinants of health concludes that “Belonging to a social network of communication and mutual obligation makes people feel cared for, loved, esteemed and valued. This has a powerful protective effect on health” (Wilkinson & Marmot, 2003, p. 22). This body of evidence points towards promotion of connectedness and social inclusion as an important way of helping people with mental health problems to rebuild a satisfying and worthwhile life. This brings us to the perspective of mental health recovery.

The recovery movement developed from within the survivor movement in the 1960s and 1970s as a critical and liberating response to the way psychiatric treatment was provided. Today, recovery has developed into an overarching aim of mental health care in large parts of the Western world (Slade, Adams, & O’Hagan, 2012). It represents a paradigmatic shift in focus for mental health services, “from its current focus on treating illness in order to produce clinical recovery, to a new focus on supporting personal recovery by promoting well-being” (Slade, 2009, p. 3). Hence, mental health recovery does not refer to full symptom remission as in a traditional psychiatric terminology (Liberman, Kopelowicz, Ventura, & Gutkind, 2002), but is defined as “a deeply personal, unique process of changing one’s attitudes, values, feelings, goals, skills, and/or roles… [and]… a way of living a satisfying, hopeful, and contributing life, even with limitations caused by the illness” (Anthony, 1993, p. 7). Symptom reduction is not considered to be unimportant but as being subordinate to personal and social aspects of recovery. In this understanding, mental health services may contribute to a person’s recovery process but recovery implies “a ‘bottom up’ approach to service development, as it begins with the needs, preferences, and goals of the person in recovery” (Davidson, Tondora, Lawless, O’Connell, & Rowe, 2009, p. 33). In the midst of these divergent perspectives, there is a lack of consensus on what recovery means in relation to mental health care delivery (Davidson & Roe, 2007; Pilgrim & McCranie, 2013). This has, in turn, been linked to sceptical attitudes towards recovery where it has been suggested that it may lead people towards failure by raising false hopes and that it entails a devaluation of professional expertise^2^ ^2^A list of “The ten top concerns about recovery encountered in the system transformation” can be found in Davidson et al. (2009, pp. 62–88). (Davidson et al., 2009; Slade et al., 2014). Furthermore, there is also a tension between views of how the demand for agency and matters of risk management should be negotiated (Maguire & Merrick, 2013; Pilgrim & McCranie, 2013).

As people with mental health problems experience stigma, disempowerment, and social exclusion, processes of recovery are also closely interlinked with social processes of change (Jacobson & Greenley, 2001; Knight, Wykes, & Hayward, 2003; Onken, Craig, Ridgway, Ralph, & Cook, 2007; Repper & Perkins, 2003). Hence, recovery is built on a rights perspective, implying “that people with mental health problems have a right to participate, as equal citizens, in all the opportunities available within the communities of their choice” (Repper & Perkins, 2003, p. x). Furthermore, there is a structural level of civil rights attached that addresses society’s responsibility for distributing equal rights to citizenship (Davidson, O’Connell, Tondora, Styron, & Kangas, 2006; Onken et al., 2007). Two extensive literature reviews on recovery and mental health problems (Leamy, Bird, Le Boutillier, Williams, & Slade, 2011; Tew et al., 2012) identified three areas significant for social recovery^3^ ^3^Leamy et al. (2011) included two more areas, “hope and optimism about the future” and “finding meaning and purpose in life”, but these are related to social recovery to a lesser degree.: empowerment and control over one’s life, connectedness, and rebuilding positive identities. These three aspects will be used as the framework for discussion later in this text.

To provide a recovery-oriented service and work towards social inclusion and citizenship in a psychiatric intensive care unit, such as that where Marco was admitted, is particularly challenging (Buckle, 2005; Dix, 2005). Rigid structures and strong focus on pathology have been found to reinforce passivity and promote hopelessness and powerlessness in such settings (Chen, Krupa, Lysaght, McCay, & Piat, 2013), and high levels of boredom and dissatisfaction with available activities have been reported (Antoniou, 2007; Healthcare commission, 2005). Recovery-promoting roles of mental health professionals have been studied and outlined, although mainly in community settings. Davidson et al. (2009) argue that in general the role of professional helper must be orientated towards supporting and facilitating the person’s own efforts through an empowering practice focusing on hope, self-determination, and community inclusion. In relation to acute and intensive care, Chen et al. (2013) add that a recovery-enabling framework for inpatient providers must promote strengths and skills as well as network building, and prepare patients for the next step of the recovery process (p. 110).

In the last decade, music therapy has been connected to several of the same theoretical underpinnings that characterise the recovery perspective such as empowerment (Procter, 2002; Rolvsjord, 2004), well-being (Ansdell, 2014; Ansdell & DeNora, 2012; DeNora, 2013), social capital (Procter, 2004, 2011), anti-oppressive practice (Baines, 2013), resource-orientation (Rolvsjord, 2010; Ruud, 2010), agency (DeNora, 2000; Ruud, 1998, 2010), and community-orientation and community music therapy (Ansdell, 2002, 2005, 2010a, 2010b; Jampel, 2007; Stige, 2002; Stige & Aarø, 2012). It has been argued that music therapy in general reflects some of the core principles of the recovery approach (e.g., Chhina, 2004; Eyre, 2013; McCaffrey, Edwards, & Fannon, 2011). However, I suggest that music therapy approaches within a humanistic, resource-oriented and community-oriented tradition most closely align with the theoretical foundation of the recovery perspective (Solli, 2012; Solli, Rolvsjord & Borg, 2013).^4^ ^4^The reader may note that also with regard to resource-oriented perspectives there are differing understandings to what degree a resource-oriented practice differs from other music therapy practices (Pedersen, 2013; Rolvsjord, 2007, 2010).


While mental health services are developing towards a recovery-oriented practice, there is a growing demand for development of “more comprehensive clinical approaches that focus on a person’s positive health, strengths, capabilities and efforts towards recovery” (Herrman, Saxena, & Moodie, 2005, p 137). Some authors have also pointed towards the need for more research to help identify the contributions that mental health services can make to recovery (Anthony, 1993; Davidson, 2003; Le Boutillier et al., 2011; Leamy et al., 2011; Slade et al., 2012). As knowledge about how recovery is promoted or hindered primarily originates from first hand-accounts of people with mental health difficulties, a qualitative methodology is seen to be particularly well suited for investigating such processes (Davidson, 2003; Davidson, Ridgway, Kidd, Topor, & Borg, 2008; Gill, 2012). A recent review of the music therapy literature identified relatively few qualitative research studies exploring service users’ experiences with music therapy (Solli et al., 2013). However, a meta-synthesis (Solli et al., 2013) and a multiple case study (Solli & Rolvsjord, 2014) demonstrate that users’ experiences regarding music therapy resemble many of the recognised benefits of a recovery-oriented practice, including facilitating such aspects as social belonging, participation, and contact with others. However, processes of mental health recovery in music therapy in an inpatient setting have not previously been explored through a single case study.

The primary purpose of the present study is to expand the body of knowledge by exploring how music therapy was experienced by one man with psychosis admitted to a psychiatric intensive care unit. By means of a qualitative single case study featuring thick description of a music therapeutic process supplemented with first-person accounts of the participant’s subjective experiences, the aim is to understand better the possibilities of music therapy as a recovery-oriented practice.

## Methodology

In line with current trends in recovery research, this study will use a qualitative methodology to explore subjective experiences, search to understand the active role of the individual in processes of recovery, and elaborate the characteristics of music therapy as a recovery-oriented service (Davidson et al., 2008). The current case was selected from nine cases reported in a larger study (Solli & Rolvsjord, 2014) exploring how inpatients diagnosed with psychosis experienced music therapy, using interpretative phenomenological analysis (IPA) (Smith, Flowers & Larkin, 2009). In this study, four superordinate themes were identified: freedom, contact, well-being, and symptom relief, each with several subthemes (Solli & Rolvsjord, 2014). The intention of the present study was to focus on one of the themes – contact – by purposively selecting a case that was expected to offer insight into the research question (Smith et al., 2009). Hence, this single case study is instrumental in its nature (Stake, 1995). The chosen single case study design provides possibilities for further elaboration and detailed exploration of the theme that represents something more than simply zooming in on previous findings (Stake, 1995).

### The participant

The participant was recruited from the present researcher’s own music therapy practice at a psychiatric intensive care unit. The “mother-study” included patients who were diagnosed with a psychotic illness and/or had experienced psychosis within the past year, who were motivated to participate in music therapy, and who were verbally capable of expressing themselves in an interview. During periods of acute psychosis, the participant’s engagement in the research project was put on hold and not resumed until the acute phase was over.^5^ ^5^These decisions were taken by the ward psychiatrist in collaboration with the present researcher, based on their own observations and/or information from the clinical team. From a hermeneutic position, the double role as therapist and researcher can be advantageous as it affords a closeness to the context and person that give access to rich data and thick descriptions (Alvesson & Sköldberg, 2000). However, this double position called for systematic and reflexive work on attitudes and positions throughout the project, as well as an ability to reflect critically on own participation (Finlay, 2003; Stige, Malterud, & Midtgarden, 2009).

The data material on which this case study was based included two qualitative semi-structured interviews (Kvale & Brinkmann, 2009), participatory observation (Fangen, 2004) documented in electronic hospital notes and a logbook after each session, and recordings of musical interplay. The interviews and the improvised raps (freestyle) were transcribed. It was hard to find time for interviews due to the participant’s lengthy periods of acute illness. Due to this, some parts of the case presentation contain quotes from interviews, while some themes comprised the researcher’s own narratives about the process. The rap lyrics from the CD were not subjected to analysis because these songs were shared in social settings outside music therapy and hence citations would threaten confidentiality.

### Analysis

Data were analysed using the flexible guidelines of IPA (Smith et al., 2009). However, this study diverges from a traditional IPA study in that the findings are presented in a narrative form rather than as a taxonomy. IPA was originally formulated to analyse transcribed interviews, but can be adopted for analysing other kinds of data, such as observation (Larkin & Griffiths, 2002). The analysis included the following steps.

First, the transcripts of the interviews and the improvised raps were read and re-read while interesting and significant sections were commented upon using the software Atlas.ti (Atlas.ti; http://www.atlasti.com). Notes from the previous analysis of the interviews were retained but revised and augmented. Second, the log from the observations was studied in the same way as the interviews and notes were handwritten in the margins. Third, through engagement in an interpretative relationship with the transcripts and the initial notes, a structure emerged, reflecting the development of the music therapy process and the participant’s reflections on this process. Since the underlying aim was to explore the user perspective, the first-hand accounts of Marco were given primacy in the analysis.

The observational data primarily served to identify the development of the therapy on a structural level and to supply the case with the level of detailed information that could only be provided by such thick description. It was important to preserve reflexivity in regard to the interpretation and “coding” of the observational data in the previous process of observation and writing while the idiographic data from the participant had a more immediate and transparent character (Larkin & Griffiths, 2002). The fact that the transcript excerpts have been translated from Norwegian to English for the purposes of publication may cause some blurring of the intended meaning from the interviews. In addition to considering data from interviews and observation, the analysis was conducted in dialogue with the study’s theoretical framework and hence had abductive qualities (Alvesson & Sköldberg, 2000).

The written presentation of the case was conducted in a narrative mode, and the subjective stories of Marco and the observational data were reconstructed with regard to the main points desired to be communicated (Kvale & Brinkmann, 2009, pp. 286–287). The study takes a critical stance towards traditional psychiatric treatment of people with severe mental illness and can hence be linked to a tradition of value-laden research (Anderson, 2012). Hence, the present study intends to look critically at the stories that are told about music therapy (Ansdell, 2003) and to contribute to develop mental health services in a more humanistic and recovery-oriented direction. The study was approved by the Regional Committee for Medical and Health Research Ethics and reported to the local health and social services ombudsman. An informed consent form was signed by the participant. An administrative nurse from the current hospital read and gave feedback on final version of the text to ensure that matters of confidentiality were dealt with appropriately.

### Music therapy context

Music therapy was an integrated part of the hospital’s multidisciplinary treatment programme. On a daily basis, the music therapist worked together in the multiprofessional treatment team which included nurses, physiotherapists, occupational therapists, social workers, psychiatrists, and psychologists. Specific therapy combinations generated shared foci and together with physiotherapy and occupational therapy, the music therapy shared a specific focus on activity, creativity, well-being, social gatherings, and the establishment of contact with services outside the hospital.

The hospital’s music therapy services consisted of individual sessions and open groups in addition to a contextual focus on promoting the hospital milieu and possibilities for connecting patients to municipal music activities (Solli, 2009). The practice was informed by ideas accumulated in humanistic, resource-oriented and community-oriented music therapy (Aasgaard, 2002, 2004; Rolvsjord, 2010; Ruud, 2010; Stige & Aarø, 2012). As a consequence, the patient’s musical identity, interests, recourses, and their desired forms of musical collaboration formed the basis of the therapy, and goals were worked out together with the patient. It also included investing effort in creating social arenas for interactions between patients (and staff), through supporting patients to collaborate musically within the frame of individual sessions, and creating arenas for performances by arranging social gatherings at the hospital. Active and structured forms of music making were dominant features in the music therapy provided, and improvisational use of groove-oriented music and popular genres were commonly used (Aigen, 2002, 2013; Solli, 2008). Marco was offered a weekly 60-minute individual session in the music therapy room and he participated in a weekly open music group on the ward.^6^ ^6^For pragmatic reasons, this group will not be focused upon in this case presentation.


## Case presentation

### Marco

Marco was just a teenager when he was admitted to a psychiatric hospital and diagnosed with psychosis for the first time. Six years later I met him at the hospital where I was working as a music therapist. During these 6 years Marco’s life had been a revolving voyage between various psychiatric hospitals and mental health centres. Marco spent most of his first weeks at the hospital inside his room lying in bed, giving little verbal response. However, the little he did say revealed the scenario of an inner battle of life and death. In a later interview he explained:

Psychosis is…kinda something that keeps me imprisoned and controls me and makes it possible for me to get lots of trouble and paranoia…Kinda threatening feelings which…just as if everybody is threatening me.

He told me that the voices were less of a problem, while the real fight was against forces that wanted to drag him into the abyss: “Yeah, it’s some demonic shit that wants to break me down, you know.” Marco was convinced that the only thing he could do to battle the dark forces was to stay focused on the good and positive aspects of life. He held onto his hopes and seemed convinced that doing positive things and participating in positive life events would help him win this war.

Those things … It’s me who’s going to win … Just by saying that, I am winning. The so-called demons, they lose, they lose, they cannot break me. (…) I just try to relax. It just gets tiring to fight. But in a way I still have to fight. I kinda have to go outside and sense these things that are trying to break me and grind me down. I might have to go on for a while. But I’ve got through the worst, then. (…) I kinda know that the war will soon be over… [I] believe that it’s going to be better and better. (…) One cannot live without things getting better and better.

Marco’s ups and downs came in waves with periods of greater levels of well-being and social activity but also days where he did nothing but stay in bed, being in a terrible and fearful condition, seemingly losing contact with the world around him.

Marco had a huge interest in music, especially rap music. He had a thorough knowledge of artists, traditions, and styles, and he had fairly good rapping skills. His interest in music was a huge resource of meaning, hope, and motivation in his life. He had a strong identity as a rapper, and this seemed to be an empowering force in his life:

… it’s interesting then – that if you are a musician, a rapper, then you have many thoughts and ideas and understanding and experiences and wisdom, so you can … Then you have much more to give, then. So if I am to be a rapper, then I can be a kind of rap-intellectual. I have many ideas about how to come up with something.

Marco had hopes and dreams about becoming an artist and being discovered by the music industry. He often said that he wanted to be a positive rapper with positive messages, criticising rappers who communicated negative thoughts and violent attitudes. At the same time he also conveyed ambivalence towards these thoughts: his dreams about becoming famous were contrasted with thoughts about rap being a meaningful spare-time activity, a precious source of fun and sociability in his life.

### Music therapy sessions

I always invited Marco to suggest activities and agendas for our sessions. In the initial weeks he preferred to explore the various instruments in the music therapy room, play prewritten songs and improvise together with me. As he worked his way through most of the instruments, rapping became an increasingly central component of our collaborative musicing. We gradually developed our own structure of improvising together. Marco started beatboxing^7^ ^7^Beatboxing entails producing drum beats and musical sounds with one’s mouth, often used in hip-hop and rap music. a beat for me to copy on the drums, and as soon as he heard me picking up the groove correctly, he would start freestyling, putting together improvised rhyming rhythmic sentences with lyrics about issues that concerned him that particular day, often mixed with some prewritten material. In one of the interviews he described how he experienced flow within this way of making music together:

It kinda flows, you know … When I’m alone, it doesn’t work out at all. But when I’m here, then, there’s always something that I can get out. Cause here I’m open. Inspiration and energy seeps out of me. It’s sort of open. I just start, and then it comes out. It’s always something… Then I’ll rap a bit, then I sing a bit, and….The thing is, I guess…. We sit here. Eh … it’s dialogue. And then… and then there’s lots of … There’s contact between me and you right now. That’s how it is with the rap too. When you’re sitting there at the drums, then there’s something, it’s kinda something … I’m rapping, right? If you’d left the room, then the rap would have disappeared, really. But when you come in here, then it’s there, right?

Marco’s mental health problems were among the most severe of any patient in our department. Hence, I was often stunned by how well he was doing in music therapy, how many musical resources he was able to draw on, and how he could work in such a structured and concentrated way in his music. His “level of functioning” always seemed to be significantly higher in music therapy than that reported from his life on the ward. I asked him about his thoughts on this in the interview:

Marco: There’s a freedom from all possible illness, then… and psychosis and everything that’s bothersome. There’s something good and creative in this room.

HP: Did you notice that when you came into the room now?

Marco: Yeah, I mean I’ve always noticed that it’s good to be here. It’s peaceful, you know. It’s peaceful in this room. There’s no disease in this room. There are no negative spirits here, somehow. There’s peace.

HP: Yes… How come it’s like that, do you think?

Marco: I guess it has to do with …you know…what’s in this room… It’s got such a value…and…what you do…then…is kinda…it’s kinda music…and music means so much in life…and when you see these things [points at the instruments] they radiate music and joy and such.

On a few occasions, when he seemed to be in a condition of acute psychosis and could do nothing but stay in bed, he said that he did not want to have music therapy. On those days I visited his bedside at the time of our sessions, but as it seemed that he needed rest and sleep more than anything else, my visits lasted no more than a few minutes.

Despite his mental challenges, Marco was often communicating a desire for togetherness with other people, especially in less critical phases of his condition. His rationale for this was connected to his experiences with flow:

Say, if I’d been alone in my room somewhere trying to rap, then it would turn out more like: ‘Yeah, I’m sitting on a chair, and … ji, ji, jia…’ And then just… but…and then…then nothing much would come out. I wouldn’t have the desire. But when I’m with another person, then flow is possible. (…) Then loads [of words] are just coming. *That’s* flow. *That’s* flow, you know.

He asked me several times whether he could bring fellow patients or friends (from outside the hospital) with him into music therapy sessions. I was reluctant to allow friends from outside the hospital to join us, mainly because this was not common practice at the hospital, but also because I often judged Marco’s condition to be too acute. On a couple of occasions Marco was allowed to bring a fellow patient from the ward. The success of this was variable as Marco showed a more tense and silent side when put in situations where other people came up with creative ideas that did not always match his.

HP: You’ve said many times that you wish you had someone to rap together with.

Marco: Yeah, I can maybe rap together with Anthony [fellow patient] then. But he’s very demanding…he wants me to write lyrics and make beats, make songs. And I’m not very motivated to create anything for the time being.

HP: No…

Marco: ‘Cause, why should I? It won’t be any good if you have to push yourself to … ‘You have to do some Jay-Z or Snoop Dogg’. They tell you that ‘You have to produce, you have to produce. The record company says you have to produce….

However, Marco’s musical collaboration with other patients in the ward provided him with a sort of social network to which he often referred in our sessions: being a musician who had relationships to other musicians became a strengthened identity, and something he could bring with him to the social world of the ward:

I like a bit of attention, and I like talking to the boys, talking about things … (…) to have boys of my own age who share the same interest. For example, it’s always cool to see George [a male nurse]. You know who George is? He’s only one year older than me. He’s also into rap. So we have a friendly tone, you see.

In what follows, I will highlight three episodes from Marco’s music therapy which were particularly significant in respect of the themes *well-being* and *sociability*: The CD, the Concert and the World Wide Web.

### The CD

One day Marco brought a sheet of paper with self-written rap-lyrics on it as well as some music files he had made on his computer, telling me that he wanted to make a CD. This was the start of a project that brought a new structure to our sessions. In what became a creative co-production of a CD, we started to alternate the roles of musician and music studio operator. Sessions often started with Marco transferring his music files to the studio computer and playing it out loud on the sound system. Then we would discuss how to work with the song – which energy and feel to emphasise, which instruments to use, the structure of the song, and so on. At this point we would switch seats again so that Marco could do the rapping while I handled the recording studio. Then if he wanted me to play live drums or other instruments on top of this, we switched seats again. Marco demonstrated a clear consciousness about the connection between his emotions and the music that he used in the recording process.

There are tones for every mood. And then we can express ourselves through music. If you play the piano, then you try to hit the notes that kinda relate to how you feel.

Eventually we recorded the five songs that were intended for the CD. In the final phase of the process, we mixed and mastered the audio files, established the order of the songs, and designed and printed the cover and label. Marco wanted 15 copies so that he could give them to fellow patients, friends, and family. The same day I made sure that the staff on Marco’s ward became aware that he had a “brand new CD out”. The next day I heard that his songs had been played on the stereo in the ward living room with many people sitting down to listen. In the following session, I asked Marco how the response to the CD had been. He reported with a smile on his face that he received lots of good feedback from fellow patients and staff members, and that he needed another 10 copies to meet the demand.

### The concert

About a month after the CD was released, the hospital was planning one of their annual seasonal parties. This was a collaborative event where patients were engaged in various art projects and a cooking group, or to contribute with some kind of entertainment. I asked Marco how he felt about performing one or two of his songs at this event, as a warm-up to the professional band that had been hired for the occasion. He responded with enthusiasm and immediately started suggesting songs to perform. He had rapped in front of an audience a couple of times before during previous hospital admissions, and the communicative aspect of rapping in front of an audience was something he valued.

If we’re talking rap… music. It’s really talking. It’s basically a dialogue between the audience and the rapper. I think that’s what many rappers do wrong…because they don’t understand, they only rap a lot of stuff, but it’s … The thing is that they have to say something to the audience. Rap is talking -talking with rhyme and rhythm.

We used the next four sessions to prepare for the performance, rehearsing the songs with playback and live drums. The details of the performance were discussed – what to expect, how to handle nerves, and the possibilities of withdrawing if it turned out to be a bad day for Marco. On the day of the concert, Marco and I met for the sound check a couple of hours before the party started. He did not seem particularly stressed, telling me instead that he was eager to play. When the time of the concert arrived, Marco performed the two songs with considerable passion and energy, even putting in some unexpected freestyling at the end of the songs. From my position behind the drums I could see many smiling faces as well as people dancing, clapping, and cheering energetically after each song.

Marco’s performance was followed by a concert by the professional band. Marco had been watching their sound check and had talked to some of the musicians earlier that day, and I could see that he was paying close attention to their show. Before the last song the lead singer suddenly asked if Marco wanted to join them on stage. Marco was not resistant to the invitation, and a moment later he was jamming together with the band, freestyling lyrics on top of the groove of some of the most experienced musicians in town. The audience was dancing, the musicians were smiling, and Marco seemed to be having the time of his life. After the show he received a great deal of attention from both staff and fellow patients, all of whom seemed impressed by his performances. Marco seemed to be smiling all night long.

Marco and I spent the next couple of sessions debriefing after the concert, talking about our experiences. He said that it had been a good experience, and that he had enjoyed the attention he had received. Here it became very clear to me how important it was for Marco to meet and be together with other people and how vital music was in this project. This resonates with what he said in the interview:

But the thing is that along with other people, then… the thing is … uh … it’s best to have music … therapy, say, along with other people. It’s positive to cooperate. It’s positive. It’s positive. That’ll be positive for all the people you’re going to bring down here later. It’s positive for them. People help…That people work together for something…instead of keeping on alone.

### The World Wide Web

A few weeks after the concert, Marco came to music therapy with a new request: he wanted me to help him to upload his music to an open access site on the Internet. During the next few sessions, we sat down together in front of Marco’s laptop and created a user account and a profile, and uploaded one of his songs. As we looked at other profiles, we noticed that users could comment on each other’s music, and Marco was curious whether anyone would listen to and offer comments on his music.

The next few weeks became quite stressful for Marco. He constantly checked the status of his profile and started to despair at the lack of response. People had evidently been listening to his song but that made him even more confused – why did they not respond? He found this hard to understand. I tried to calm him down as best I could and explained how hard it is for anyone to get attention in today’s crowded music culture. Marco eventually started to say that he did not care and that he knew his music was good enough anyway, but I could see that he was still very disappointed.

Then one day he came to music therapy and was very upset. He had received a response from a girl who had listened to his song, and she had written that she did not like his music at all. She even wrote that his rap did not flow and questioned his seriousness as a rapper. Marco responded with great irritation and anger, at the same time telling me (and himself) that he did not care because music was not that important to him anyway. However, the girl and her comments were all he could talk about in our sessions. I then asked him how he might feel about formulating his frustration in a musical way. I encouraged him to freestyle a rap about how he felt without holding back. In an imaginary battle rap, together with me on the drums, the following rap came out^8^ ^8^In the original Norwegian, this rap has rhymes in almost every sentence, something which is lost in the translation.:

I don’t give a fuck, I know I’ve got skills. Hah! So I rap stuff, and this hasn’t got no meaning, but it doesn’t matter ‘cause I’m getting better every day. This ain’t no serious rap, it ain’t no crap either. Whatever, I get my rap through – that’s all that counts. Just wait and I’ll get it out, yo. I’m just waiting for that flow to come. I’m just waiting for the rap to come … then I’ll make you gape. And you who say I’m shit, you’re a fuckin’ dumb ass. What the fuck. I won’t take no more shit. Haha. I’m not serious … ‘cause I’m not lacking inspiration. But I’m neither serious nor lacking inspiration. So I’m enjoying my rap, while you’re trying to be a great rapper, what the hell, while I’m sitting here being a TV zapper. I’m having fun with the rap. I meet lots of other rappers – you don’t meet any of them. What the hell. Haha. So I don’t care – but I can still be your friend. Do some rap together with you. I’m not thinking ‘bout playing concerts to get rich. You may say I’m crap at rapping, but you’ve got no fuckin’ reason to say that. You’ll have to beat the bullet. Sooner or later, woman, you’ll have to start crocheting! Haha! You‘re no rapper. I’m dissin’ you! Hah! (…) I don’t give a fuck. This is freestyle. Hell, I’m a real rapper and I’m no shit!

This rap was one of the last things Marco and I did together before he was discharged and moved to another mental health institution. I burned all his songs and freestyles onto a CD so that he could take it with him – as memories of what we had done together, but also as inspiration and motivation for recordings, performances, or uploads in the years to come.

## Discussion

The purpose of this study was to explore how music therapy afforded one man in a psychiatric intensive care unit possibility for social recovery. Marco’s experiences with music therapy, as described above, can be connected to several aspects important for social recovery. In the following, I will highlight three areas that are found to be important for social recovery both in studies of music therapy and recovery (Solli & Rolvsjord, 2014; Solli et al., 2013) and in the recovery literature, in particular that inspired by the work of Leamy et al. (2011) and Tew et al. (2012): agency and empowerment, (re)building a positive identity, and connectedness.

In order to illuminate the research question indicated, I will apply the concept of affordance and appropriation as described by DeNora^9^ ^9^These terms were originally conceptualised by James J. Gibson (1979/1986). (DeNora, 2000, 2007). DeNora offers an alternative to the traditional approach of looking for the effectiveness of a therapy in the intervention itself. Instead, she frames health as a relational, emergent, and collective unity that is performed by the person. In this understanding, the effect or meaning of music is dependent on how it is used or acted upon. An activity such as playing music affords health promotion only if it is used, or appropriated, in ways that promote health, to put it simply. Hence, the notion of affordance and appropriation supports the recovery approach’s fundamental view that recovery is an active process in which the person is the true agent. This perspective is also reflected in the notion of “client’s craft” in music therapy (DeNora, 2006; Rolvsjord, 2010).

### Agency and empowerment

Mental health difficulties are associated with disabling social situations of powerlessness, discrimination, and social exclusion – processes which can be further amplified by paternalistic services and treatment (Tew et al., 2012). To avoid such negative processes, it is important that people with mental health problems become *active agents* in their own recovery process rather than passive receivers of treatment and care (Davidson et al., 2009; Deegan, 1996b). In relation to recovery, agency can be defined as “the ability to view oneself as a person capable of choosing, initiating, doing and accomplishing things in the world” (Slade, 2009, p. 197). Such a view calls for a practice where goals and methods are products of collaboration between the patient and therapist, in accordance with principles highlighted in resource-oriented music therapy (Rolvsjord, 2010). In line with this, Marco was always invited to come with suggestions and ideas about what music therapy should be about and how to do it. His suggestions and wishes laid the ground for a rich bouquet of opportunities for musical and social participation. Small (1998) offers the term “musicing” in order to call attention to the active and performative ontology of music, and how music engages people in a variety of roles. In the case of Marco, music therapy afforded musicing connected to roles as a *musician* (rapping and playing diverse instruments), *songwriter* (who composed music and wrote lyrics), *producer* (in charge of the recording process), *technician* (operating the mixer and the software), *manager* (with responsibility for planning parts of the performance), *merchant* (distributing CDs to interested people), and *promoter* (making his music accessible on the Internet). Managing all these roles included processes of choosing, initiating, doing, and accomplishing things, and would arguably promote Marco’s sense of agency.

A mutual partnership between professionals and patients, one which acknowledges the power of both expert positions, is found to be important for promoting recovery and empowerment (Boevink, 2012; Borg & Kristiansen, 2004; Onken et al., 2007; Slade, 2009; Wallcraft et al., 2011). In the recovery literature, people with experiences of mental health problems are referred to as *experts by experience* who hold valuable and unique knowledge about their own life history, their interests and strengths, and strategies influencing their recovery processes (Slade, 2009). Nobody knew Marco’s previous life experiences better than he himself. Although he often isolated himself when having bad days, he seemed to know that he was in need of positive life events and social contact – strategies which perhaps previously he had experienced as helpful. Musically, he had fine rap skills and a much greater knowledge of artists and records in the genre of rap and hip-hop than his music therapist. This resource could be argued to render him *expert on own music*.

Music therapists are experts through their professional knowledge about music and health and the possible relationships between them (Stige, 2002). They are trained to be emotionally and musically attuned to other people, to adapt musical participation to levels of skills and functioning, and to control contextual factors in order to provide structure and predictability. The interaction between the Marco and myself implied a flexible movement in and out of each person’s area of expertise. When Marco instructed me how to play the drums by beatboxing rhythms into the microphone or told me about a new CD release of his favourite rapper, he was in the “driver’s seat”, while 10 minutes later, as he wanted me to show him some new chords on the guitar, I had to use my expert competence. In addition, the joint musicing afforded mutuality in our relationship: as Marco laid down the rap and I provided a drum beat, the quality and flow of the performance relied on the two of us being mutually aware of each other’s contributions in the present moment, getting into the groove together (Aigen, 2002; Solli, 2008). I think one of Marco’s improvised rap intros describes well the nature of the relationship that developed between us: “Yo! I’m just doin’ some shit with my man Hans Petter on the drums. Yo! He’s playing the drums and I know he won’t just do it for getting the money.” This statement points towards the establishment of a partnership relationship (Slade, 2009), a “meeting of equal minds” (Ansdell & Meehan, 2010, p. 34) where two experts could share their competence in the music room.

It can however be argued that the relationship between a patient and a therapist is far from mutual. As a therapist at an intensive care unit I was equipped with keys to lock and unlock doors. I had a visible alarm in my belt in case of emergency. I reported all our sessions in the electronic patient record, and I was a part of a multiprofessional team which practised compulsory treatment – and of course I did get paid doing my job. All these aspects could conventionally cause a huge imbalance of power in the relationship between Marco and myself. However, in spite of these obvious inequalities of power, it seemed that music therapy was an empowering arena for Marco. He seemed to gain power by participating in the musical interplay and his musical agency flourished: “Inspiration and energy seeps out of me.” This could be understood in relation to the nature of musical interaction itself as a source to experiences of mutuality (Ansdell, 2014; Ruud, 1998). Additionally, from recovery research we know that it is important that professional helpers have a strong focus on patient’s strengths, interests, and positive identity to counteract experiences of powerlessness and reducing stigma (Slade, 2009).

Lysaker and Leonhardt (2012) highlight the interpersonal and communicative aspects of agency and argue that being an agent “is the result of the recognition and basic experience one has at an elemental bodily level which can be shared with and understood by other people” (p. 165). Music was a medium where Marco could take the role of the narrator (loudly, via the microphone) and share his stories and worldview with other people – first with the music therapist and then with a greater audience through the CD, concert, and on Internet. Hence, playing music afforded many possibilities for communication of agency, both through the activity he was doing (being a rapper and performing his music) and through his lyrics (where he demanded a good life despite his challenges).

Empowerment as process is a multilevel construct comprising personal, interpersonal, and political levels (Gutiérrez, 1990). From a user perspective, Deegan (1997, p. 11) understands empowerment as “the struggle to take back that power, to become sovereign over our own lives and bodies, to reclaim our right to make choices and to have access to resources to improve the quality of our lives.” The above elaboration of how music therapy afforded Marco agency and expertise can be understood as a means for him to gain power to take action to improve his life situation, on personal and interpersonal levels. Hip-hop and rap music is commonly used to express feelings of suppression, to criticise repressive social conditions and claim the right to power (Tyson, 2006; Veltre & Hadley, 2012). As Marco was so disturbed and reduced by his psychotic experiences, the rap was not so much about outer social conditions, but rather about battling his inner “demons” and rebuilding and expressing hope for better days to come. However, as the different levels of empowerment are interrelated (Travis & Deepak, 2011), the control and power Marco gained from his musical engagement on a personal level would also be a source of support for his process of social recovery.

### (Re)building a positive identity

As mental health difficulties are often associated with an illness-dominated identity, the development of a positive identity dominated by agency, competence, and well-being has been found to be an essential part of recovery (Davidson et al., 2009, 2005; Mancini, 2007; Onken et al., 2007; Slade, 2010). Davidson et al. (2009) argue that “The redefinition of oneself as a person of whom mental illness is simply one part may be one of the most essential and overarching aspects of recovery” (p. 45). Over the years of hospitalisation, Marco’s identity of being a mental health patient had become a part of his personal identity, and his social network was to a large degree limited to staff and fellow patients. However, music and rap was important for how he thought about himself and presented himself to others. Through rap music he became aware of his positive qualities of being creative, intelligent, cool, or tough, and it was an arena where it seemed acceptable to perform self-confidence and pride. He could even diss^10^ ^10^Slang for showing disrespect. the people he disliked, as in the case of the girl from the Internet, express his frustration towards the hospital, and sing out his praise to the girls with whom he fell in love. He started to trust in his skills, believe in himself, and see possibilities in life – as he shouted out in one of his improvised raps: “I don’t care, I know I’ve got skills. …I am a real rapper and I’m no shit.” Thus, it seems as musicing afforded self-confidence, agency, and wellness which could be appropriated in the battle against his mental health difficulties and an illness-dominated identity.

The increased well-being and self-confidence Marco gained through music also affected his social life. The identity of being a rapper, which seemed to offer him a positive feeling of being a skilful person in flow, seemed to provide motivation and courage for sociability – at least in his more stable periods. Furthermore, music afforded possibilities for participation in meaningful social gatherings, such as discussing music with staff and other patients, playing in the concert and talking to other musicians, and communicating with other rappers on the Internet. Such engagement in normal activities is found to be important for recovery (Davidson et al., 2005; Slade, 2010).

It could be argued that building a personal and social identity on being a musician-rapper while having severe mental problems is akin to building a house without a foundation, leaving it fragile to the streams of life which might later reduce it to ruins.^11^ ^11^Luke 6: 46–49 (New International Version). Although Marco had fairly good rapping skills and a few contacts within the music business, the chances of his becoming a professional and make a living out of such a career were probably small. Is the idea of supporting an identity based on music skills and unrealistic dreams of becoming a famous rapper such a good idea? First of all, Marco’s positive identity of being a rapper was not invented by the music therapist but rather the product of a life-long process on Marco’s part. Ruud (1998) demonstrates how music can play a fundamental role in the construction and development of people’s identity on a personal and social level, processes also reported in music therapeutic use of pop-rock music (Aigen, 2002; Ansdell, 2005; Krüger, 2011; Solli, 2008) and hip-hop/rap (McFerran, 2012; Veltre & Hadley, 2012). The support of such identities must therefore be seen as acknowledgement of the person himself, and an essential principle in a contextual and humanistic approach to mental health care (Ruud, 2010). It is also important to note that promoting a positive and healthy identity does not mean ignoring problems or setting aside previous life experiences. Rather the goal must be to restore a balance where a positive sense of self is amplified and the overwhelming identity of being ill is diminished (Slade, 2009).

Schiff (2004) offers a first-hand account of how music, and the identity of being a classical singer, was helpful in her own recovery process. “It [singing] grounded me and connected me. It allowed me to feel human when my illness dehumanized me” (p. 216). When ill, her goal was to achieve a professional musical career, but as she recovered her plans changed: “It was not until I really had recovered (…) that I parted from music as a career. I was thankful for its help in my recovery, but I no longer needed it” (p. 216). This resonates with how Marco sometimes talked about music as all he cared for, while at other times he referred to it as a hobby that made the days go by. In this way music affords a scaffolding identity that can be appropriated by persons with mental health problems, a personal and social identity which is hope-promoting, offers a sense of belonging and is a positive alternative to an illness-based identity. Such a positive identity is especially important for the person in the process of recovery and can help to develop a resistance against stigma (Rusch, Lieb, Bohus, & Corrigan, 2006).

### Connectedness

Relationships with other people are found to be of great importance for mental health recovery. However, the quality of the relationship is vital as some types of contact may represent bad influence and can make people feel more disempowered and stigmatised (Topor et al., 2006). Supportive and hope-inspiring relationships, on the other hand, are associated with growth, development, and well-being (Repper & Perkins, 2003; Tew, 2013; Tew et al., 2012; Wilkinson & Marmot, 2003), and are found to be particularly effective when individuals perceive a shared identity with another individual or group (Jetten et al., 2014).

As we saw in relation to the development of agency, positive and mutual relationships between patients and professionals can be vital for processes of recovery. However, establishing such relationships in psychiatric acute or intensive settings can be challenging. Chen et al. (2013, p. 100) identify obstacles to the development of recovery-promoting relationships in inpatient settings to be “uncritical application of the medical model, a custodial framework, and risk-control principles”. As recovery is a process owned by the person with mental health difficulties, and the goal is to enable the person to live a hopeful and fulfilling life in the community, the best way for professionals to support this process is to strive for reciprocity and collaborative partnership (Borg & Kristiansen, 2004; Topor et al., 2006).

Although the relationship between patient and the professional can be of vital importance for processes of change, other types of relationships and contacts may be even more important for social recovery. Marco’s musical interest and participation in music therapy brought him into situations and events that afforded contact with other people. The CD project and the concert offered opportunities to connect with fellow patients, staff, and other musicians and to reconnect with friends and family. The uploading of music to the Internet had the potential to connect Marco with a cyber-world of other musicians and music lovers, and may have contributed to a sense of belonging. In these meetings, music and the related activities seemed to support the process of being social and facilitated relationship-building: as a result Marco seemed to be building up his social network. I will now consider more closely how music therapy afforded contact with fellow patients, friends, and family.

There is a growing awareness of how other people with mental health difficulties, fellow patients in our context, can contribute in the form of social support that is experienced as promoting recovery (Mead & MacNeil, 2006; Slade, 2009). Marco often wished to play with other patients, but whenever a fellow patient was brought into the therapy the cooperation turned out to be quite complicated. Reasons for the difficulties varied, but were typically rooted in a lack of relational competence mixed with a currently unstable life situation, making the sessions stressful. Nevertheless, Marco reminds us of the value of playing music together with others, not just with the music therapist. Although the sessions could be challenging, I observed that Marco stayed in contact with people he had played with and with whom he shared a musical identity with. This suggests that the development of group approaches which can be accommodated within intensive inpatient settings could be important and valuable for this group of patients, since these afford important social connections that can be helpful in processes of recovery.

The support of friends and family is an under-researched subject, but is considered to be of great significance for people suffering from mental distress (Topor et al., 2011, 2006). Marco’s family and friends played peripheral roles in music therapy, with the only point of contact being when Marcus distributed the CD to them. Marco suggested several times that he could bring a friend with him to music therapy sessions, but as this was not common practice at the hospital I was resistant to following up his suggestion. Was that a right decision? I’m not sure. Within a paediatric setting, Aasgaard (2002) describes how family members were included in various ways into music therapy for their children with cancer in a hospital setting. He illustrates how such ecological work led to enhanced support between family members and the patients. To my knowledge such an approach has not been outlined in music therapy in mental health care. It can be argued that a mental health setting is a practice with far more complicated family relationships, and that a temporary distance from family may be for the better. Nevertheless, how would it have been to have invited Marco’s parents and friends to the seasonal party to see him perform? As social inclusion has become a major goal in mental health care, I suggest that this is one aspect of practice that needs to be further considered and developed.

We have now seen how music therapy can be an arena that affords possibilities for strengthening interpersonal relationships, although not all the possibilities seem to have been fully utilised in this case. The greater goal of social inclusion is about active citizenship and the subjective sense of belonging, both strongly connected with being part of social networks and engagement in meaningful social activities (Tew et al., 2012). Such social networks and connections can be understood in terms of social capital,^12^ ^12^For a thorough introduction to the theory of social capital and its possible connections to music therapy, see Procter (2011). defined as “features of social life – networks, norms and trust – that enable participants to act together more effectively to pursue shared objectives” (Putnam, 1996, p. 34). An absence of social capital has been found to obstruct recovery, while participation in meaningful activities offers (re)building of relationships and experiences of togetherness and belonging that support recovery (Tew, 2013). Procter (2011) argues that music therapy might not result in social capital directly, but rather promote what he calls *musical capital*, a type of proto-social capital that has a reparative function on communicative skills that can indirectly promote participation in society. In Marco’s case, the promotion of communicative musicality was undoubtedly an important aspect of the therapy. However, I would argue that music therapy also resulted in real social capital as Marco’s social network did in fact expand. Fellow patients, the staff, his family and friends, the girl on the Internet – this group of people formed a small and loosely connected network of people with whom he could share his music, from whom he could receive feedback, and in relation to whom he could feel a sense of belonging. Contact between people with mental health difficulties (peer relationships) is underrated as a resource and is reported to be positive for many people’s social lives and recovery (Slade, 2009). However, it is also important to notice that extensive networks are not preferable for some people with mental health challenges (Boydell, Gladstone, & Crawford, 2002).

Tew (2013, p. 369) identifies *motivation* as a premise for social capital, suggesting that affordance is not enough if the motivation to appropriate it is not present. In Marco’s case, music therapy was experienced as an enjoyable and motivating activity, and even more enjoyable and motivating when being shared with others. Such interlinked processes of well-being, motivation, and social participation are also found in other studies (Rolvsjord, 2010; Solli & Rolvsjord, 2014). Hence, it can be argued that music therapy is a promising approach and arena for social recovery for people with mental health difficulties.

### Concluding remarks

Marco was battling his demons, his psychosis, and the girl who called him a lousy rapper. But more than battling the negative forces, he was embracing life and its possibilities for well-being and sociality in ways that should be inspiring for us all. Music therapy was an arena where he could engage in processes of strengthening agency, (re)building identity, developing positive relationships, and expanding his social network. The case also illustrates how music therapy has the potential, even in inpatient settings, to help build bridges from a hospital setting to other social and cultural arenas. Since recovery-oriented services in inpatient settings have been relatively little researched or developed practically, I hope the present study can be a contribution in this direction. One of the most influential authors in the recovery literature, Pat Deegan (1996b, p. 2), drawing on her own experiences of living with mental health difficulties, suggests that, rather than asking what is wrong with people, we need to start asking “How do we create hope-filled, humanized environments and relationships in which people can grow?” The present case study is one example of how music therapy can afford such an environment for the building of such relationships.
